# Impact of an Education-Based Antimicrobial Stewardship Program on the Appropriateness of Antibiotic Prescribing: Results of a Multicenter Observational Study

**DOI:** 10.3390/antibiotics10030314

**Published:** 2021-03-17

**Authors:** Federica Calò, Lorenzo Onorato, Margherita Macera, Giovanni Di Caprio, Caterina Monari, Antonio Russo, Anna Galdieri, Antonio Giordano, Patrizia Cuccaro, Nicola Coppola

**Affiliations:** 1 Department of Mental Health and Public Medicine–Infectious Diseases Unit, University of Campania Luigi Vanvitelli, 80138 Naples, Italy; fede.calo85@gmail.com (F.C.); lorenzoonorato@libero.it (L.O.); macera.margherita@libero.it (M.M.); caterina.monari@gmail.com (C.M.); antoniorusso.ar.ar@gmail.com (A.R.); 2 Infectious Diseases Unit, AORN Sant’Anna and San Sebastiano, 81100 Caserta, Italy; giov.dicaprio@gmail.com; 3 Direzione Sanitaria, AOU Vanvitelli, 80138 Naples, Italy; anna.galdieri@policliniconapoli.it; 4 Direzione Generale, AOU Vanvitelli, 80138 Naples, Italy; antonio.giordano@policliniconapoli.it; 5 Direzione Sanitaria, AORN Sant’Anna and San Sebastiano, 81100 Caserta, Italy; patriziacuccaro@hotmail.com

**Keywords:** antibiotics, antimicrobial stewardship, quality of prescription, prescriptive appropriateness, surgical prophylaxis

## Abstract

To evaluate the effect that an education-based Antimicrobial stewardship program (ASP) implemented in two hospitals in southern Italy had on the quality and appropriateness of antibiotic prescription. We conducted a multicenter observational study in two hospitals in the Campania region. Only some departments of both hospitals were already participating in the ASP. We collected data on all patients admitted on the day of evaluation in antibiotic therapy or prophylaxis through a case report form. The primary outcome was to investigate the difference in the appropriateness of the antibiotic prescriptive practice in the departments that had joined the ASP and in those that had not participated in the project (non-ASP). The total number of patients assessed was 486. Of these, 78 (16.05%) were in antibiotic prophylaxis and 130 (26.7%) in antibiotic therapy. The prescriptive appropriateness was better in the units that had joined ASP than in those that had not, with respectively 65.8% versus 22.7% (*p* < 0.01). Patients in the non-ASP units more frequently received unnecessary antibiotics (44.9% versus 0%, *p* = 0.03) and, as surgical prophylaxis, the use of antibiotics not recommended by the guidelines (44.2% versus 0%, *p* = 0.036). Multivariable analysis of the factors associated with prescriptive appropriateness identified ASP units (*p* = 0.02) and bloodstream or cardiovascular infections (*p* = 0.03) as independent predictors of better prescriptive appropriateness. The findings of the present study reinforce the importance of adopting an educational ASP to improve the quality of antimicrobial prescription in clinical practice.

## 1. Introduction

Antimicrobial resistance is one of the greatest threats to global health today, leading to increased mortality, higher medical costs and prolonged hospital stays [1]. The number of estimated cases of infections with selected antibiotic-resistant bacteria occurring in 2015 in the EU and European Economic Area (EEA) was 671,689, accounting for about 33,110 attributable deaths and 874,541 disability-adjusted life-years (DALYs) [2]. In particular, among European countries Italy and Greece have the greatest burden of infections due to antibiotic-resistant bacteria [2].

Although antimicrobial agents used to treat infections are lifesaving, their overuse is one of the main drivers of antimicrobial resistance, resulting in the increased emergence and spread of multidrug-resistant bacteria. In hospitals the proportion of broad-spectrum antimicrobials used varied from 16% to 62% across Europe [3]. These drugs are not always necessary, and when their use is required, the selection, dose, route of administration and duration of treatment may be inappropriate [4,5,6,7].

To improve antibiotic prescriptions in the hospital setting is of utmost importance to avoid heading into a post-antibiotic era, where common infections and minor injuries can once again kill [1]. Thus, the implementation of programs for optimizing the use of antibiotics in hospitals is a public health priority. Antibiotic stewardship interventions are effective in increasing compliance with antibiotic policy and reducing consumption and duration of antibiotic treatment without, however, leading to an increase in mortality [8,9].

The objectives of the present study were to provide a snapshot analysis of antibiotic use and to investigate the impact of an educational antimicrobial stewardship program (ASP) on the appropriateness of antibiotic prescribing in two hospitals in southern Italy, in order to identify priority areas of intervention and implement measures aimed at optimizing antibiotic prescribing.

## 2. Materials and Methods

### 2.1. Study Design and Setting

This study was carried out in two hospitals in Naples and Caserta, in the Campania region of southern Italy. The 268-bed teaching hospital located in Naples serves adults and pediatrics in 30 units; the community hospital in Caserta has about 486 active beds in 23 units. Only the latter is equipped with an emergency department.

A persuasive-educational ASP, based on audit and feedback conducted by a team of infectious disease consultants [8], was started in January 2017 in Naples, and April 2018 in Caserta. The departments included in the ASP were those with the highest consumption of broad-spectrum antibiotics (in terms of defined daily dose) and/or those who had greater interest and willingness to join the project. In particular, five medical units (nephrology, neurology, endocrinology, geriatrics and infectious diseases), six surgical units (thoracic surgery, orthopedics, general surgery, gynecology and otolaryngology) and four intensive care units (ICUs) (of which one was a cardiac surgery ICU) were involved in the ASP. Briefly, we identified a multidisciplinary team, including infectious disease consultants, clinical microbiologists, pharmacists and a statistician. During the audits all patients on antibiotic treatment were evaluated, and recommendations on the indication, choice of antibiotics, route of administration and duration of therapies were given to the physicians in care. For each unit involved in the ASP one or more reference physicians were identified to carry out the audits with the infectious disease consultants. During the audits, carried out once or twice a week at regular intervals depending on the complexity of the unit, the adherence to these indications was evaluated. Furthermore, the infectious disease consultants were responsible for writing and sharing diagnostic and therapeutic protocols, based on the local epidemiology, for the management of the most common infectious syndromes. Moreover, all units of both hospitals had been involved since March 2019 in the “bacteremia program”: for five days a week, in the case of bloodstream infections, the microbiology unit informed the infectious disease specialists of positivity to allow better management of the patient.

The present study was prospectively planned by the infectious disease consultants of the ASPs and by the healthcare administration of the two hospitals that approved and institutionalized the ASP. They prepared the study protocol in September 2019 and a pre-formed case report form. The team of infectious disease consultants at both hospitals received the same training and education on local and international infectious guidelines to follow. They also were trained with the same methodology in conducting the ASP.

Thus, the snapshot analysis of antibiotic use and the evaluations of appropriateness of antibiotic prescribing were performed by audit in the two hospitals, respectively, in the week 10–14 of February 2020 in Naples, and 14–23 of November 2019 in Caserta. The audits in Naples were performed by infectious disease consultants operating in Caserta, and the audits in Caserta by those operating in Naples; the consultants were unaware if the units were participating in the ASP or not.

### 2.2. Data Collection

The pre-formed case report form was filled-in on the days of evaluation by the infectious disease consultants to assess antibiotic prescriptive appropriateness, both in the units that had joined the ASP and in those that had not participated in the project.

The data collected included the following: type of unit, number of patients admitted to the ward in empiric or targeted antibiotic therapy and/or antibiotic prophylaxis (type of antimicrobial, dosage, way of administration, indication and duration). For each antibiotic prescription the appropriateness in relation to the patient’s clinical state and guidelines was evaluated. We included all types of antimicrobials in the evaluation: antibacterials and antifungals for systemic use, antituberculosis, antiprotozoal and antimalarials and antivirals for systemic use. We did not include topical antibiotics.

For each patient in antibiotic therapy additional data were collected. They included the type of acquisition of infection (nosocomial, according to US CDC criteria [10], healthcare-associated or community-acquired), presentation with sepsis or septic shock [11], the site of infection and whether the therapy was empirical or targeted.

### 2.3. Assessment of Appropriateness

The appropriateness of antimicrobial treatment was defined a priori in the phase of planning of the present study (in September 2019). Precisely, treatment was defined adequate if it was active towards the pathogen responsible for infection and if it was correct in dosage, duration and way of administration [12,13]. A therapy was defined empirical prior to the identification of the germ with relative antibiogram or targeted otherwise. Empirical therapy was considered adequate when at least one antimicrobial with activity against the most frequent germs involved in relation to the presumed site of infection was administered [14].

For every antibiotic prescription deemed inappropriate, the reason for inappropriateness was recorded. Only one reason for inadequacy was enough to consider the treatment as inadequate, and it could be considered inadequate for several reasons at the same time. The antibiotic was considered unnecessary when the patient’s clinical state did not show evidence of infection. Antimicrobial therapy was considered inadequate when not indicated by the guidelines or when the antibiotic had no activity on the etiology involved [12].

Antimicrobial therapy was considered not recommended when, in the targeted treatment, the antibiotic was active but had an excessively wide range of action for the expected etiologies or, in the case of empirical treatment, it was not recommended by the guidelines. The duration of antimicrobial therapy was considered excessive when it exceeded the recommended limits of the international guidelines.

### 2.4. Outcomes

The primary outcome was to assess the appropriateness of antibiotic prescribing in the two hospitals; in particular, to assess whether there was a difference in the adequacy of prescriptive practice in the departments that had joined the ASP and in those that had not participated in the project.

The secondary outcome was to identify the main reasons for prescriptive inappropriateness in order to identify priority areas of intervention and put in place measures to optimize antibiotic prescribing.

### 2.5. Ethics

All methods used in the study were in accordance with the international guidelines, with the standards on human experimentation of the Ethics Committee of the Azienda Ospedaliera Universitaria, University of Campania, and with the Helsinki Declaration of 1975, revised in 2013. Since the leadership of the University of Campania and AORN Caserta formally approved the study, and the data were collected in the aggregate manner during routine clinical activities, no approval was required by the local Ethics Committee.

### 2.6. Statistical Analysis

Continuous variables were summarized as mean and standard deviation, and categorical variables as absolute and relative frequencies. For continuous variables, the differences were evaluated by the Student *t*-test; categorical variables were compared by the chi-square test, using exact procedures if needed.

Variables with a univariate *p* value < 0.1 were chosen for inclusion in an exploratory multivariable analysis, which was performed using logistic regression.

## 3. Results

### 3.1. Characteristics of Units Participating in the Study

A total of 53 units, between the two hospitals, were assessed; in particular, 19 medical, 25 surgical and 9 ICUs were evaluated. Of the total number of units evaluated 15 had joined the ASP and 38 had not. The total number of patients assessed was 486. Of these, 208 (42.8%) were being treated with antibiotics, specifically 78 (16.1%) were in antibiotic prophylaxis and 130 (26.7%) in antibiotic therapy.

### 3.2. Appropriateness in ASP and Non-ASP Units

The global adequacy of antimicrobial prescription and a comparative analysis between the wards that had joined the ASP units and those that had not (non-ASP units) are summarized in Table 1.

The prescriptive appropriateness was better in the ASP units than in the non-ASP units (65.8% versus 22.7%, *p* < 0.01). The inappropriateness was more frequently due to unnecessary (38.7%) or not recommended prescriptions (35.6%) or excessive duration (31%) in non-ASP units, while inadequate antibiotic prescription (21.4%) or dosing (28.6%) was more frequent in the ASP group, although the difference was not statistically significant. Patients in the non-ASP units were more frequently treated with third-generation cephalosporins (20.9% versus 7.3%, *p* = 0.01) and quinolones (17.4% versus 2.4%, *p* < 0.01) (Table S1). On the contrary, first-generation cephalosporins were more frequently prescribed for patients in the ASP units (53.6%) than for those in the non-ASP units (10.2%, *p* < 0.01) (Table S1). Only 10 patients were taking other antimicrobials such as antifungals and anti-tuberculosis agents.

### 3.3. Factors Associated with Appropriate and Inappropriate Antimicrobial Prescription

As shown in Table 2, a comparison between appropriate and inappropriate prescriptions showed that antimicrobial therapy was more frequently appropriate in ASP units (41.5% versus 9.8%, *p* < 0.01), especially in medical units (50.8% versus 28.7%, *p* < 0.01) and ICUs (15.4% versus 5.6%, *p* = 0.02).

On the contrary, inappropriate prescriptions were more frequently reported in surgical units (65.7% versus 33.8%, *p* < 0.01) due to the inappropriate prescription of antibiotic prophylaxis (43.4% versus 24.6%, *p* = 0.01) (Table 2). Moreover, appropriateness was more frequently reported in the 11 patients with bloodstream infections (*p* < 0.01) and in the 5 patients with bacterial endocarditis (*p* < 0.01); instead, the 16 patients with abdominal infections were more frequently treated with inappropriate antimicrobial therapy (*p* < 0.01). Another factor associated with appropriate prescription was the collection of microbiology specimens (*p* < 0.01), while a factor associated with inappropriate prescription was the presence of nosocomial infections (*p* = 0.04) (Table 2).

Multivariable analysis of the factors associated with prescriptive appropriateness identified ASP units (*p* = 0.02), and bloodstream or cardiovascular (*p* = 0.03) as a source of infections, as independent predictors of better prescriptive appropriateness (Table 3).

### 3.4. Analysis of Antimicrobial Therapy and of Antibiotic Prophylaxis

Considering only antimicrobial therapy, no differences in acquisition, source and severity of infection were observed between the ASP and non-ASP units (Table 4).

In the ASP units the patients were less frequently treated with an empirical antimicrobial therapy (37% versus 84.5%, *p* < 0.01), and microbiological samples were more often collected before the start of therapy (85.2% versus 48.5%, *p* < 0.01); moreover, antimicrobial prescriptions were more frequently appropriate (77.7% versus 33%, *p* < 0.01) (Table 4). Non-ASP wards more frequently gave unnecessary antimicrobials (44.9% versus 0%, *p* = 0.03) (Table 4).

Considering only the antibiotic prophylaxis, as reported in Table 5, ASP units showed better prescriptive appropriateness (57.2% versus 18.8%, *p* < 0.01), whereas, in the non-ASP units the use of antibiotics not recommended by the guidelines was more frequently observed (44.2% versus 0%, *p* = 0.04).

## 4. Discussion

Antibiotic resistance is rising to dangerously high levels all over the world. Overuse and misuse of antibiotics contribute to the acquisition and spread of infections due to antibiotic-resistant bacteria. In the present study, we report the effects that an education-based ASP implemented in some units of two hospitals in the Campania region of southern Italy had on the appropriateness of antimicrobial prescription. Specifically, we observed a higher antimicrobial prescription appropriateness for both antibiotic therapy and prophylaxis in the units that joined the ASP; moreover, a significant decrease in the use of antibiotics with a high environmental impact, such as third-generation cephalosporins and quinolones, was observed in ASP units. Programs and interventions that aim at optimizing antimicrobial use, i.e., antimicrobial stewardship programs, have grown exponentially in recent years [15], proving effective and important in hospital settings. The introduction of ASP in hospitals involves a series of structured interventions. They first include the institutionalization of the program so that it is recognized and progressively accepted by all the professionals of the hospital. Secondly, it is important to assess the local data on prevalence of antibiotic resistance and the consumption of antibiotics in order to implement tailor-made interventions.

ASPs in hospitals have shown a positive impact, with reduced length of stay, shorter treatment duration without an increase in mortality and a reduction in colonization and infection with resistant bacteria [9,16]. In our previous work [8], we observed a significant decrease in antimicrobial consumption, and in the incidence of bloodstream infections due to multidrug-resistant Gram-negative organisms, in two intensive care units after the implementation of a persuasive educational ASP. Educational interventions, although less immediate than restrictive methods, have a more sustained impact in influencing prescriptive behavior and can yield even better long-term results [17]. Antimicrobial stewardship includes not only limiting inappropriate use, but also optimizing antimicrobial selection, dosing, route and duration of therapy to maximize the clinical cure [18]. Thus, the present study confirms the effects of an ASP on the appropriateness of antimicrobial prescription since the participation in an ASP was an independent factor associated with appropriate antimicrobial prescription.

When analyzing the reason for inappropriate prescriptions, we found a higher frequency of unnecessary (38.7%) or not recommended prescriptions (35.6%) in non-ASP units as compared to ASP units (21.4% and 28.6%, respectively), while inadequate antibiotic prescriptions (21.4%) or dosing (28.6%) was more frequent in ASP groups, although none of these differences reached statistical significance. A commonly feared consequence of narrowing the spectrum of empirical therapy is the prescription of antibiotics that are ineffective against the clinical isolates. However, we should consider that in our study the definition of inadequate empirical therapy does not imply a lack of effectiveness against the isolated strain, but only against the totality of the potentially expected etiologies. Furthermore, we should point out that if we consider the total number of prescriptions, the rate of inadequacy is similar in non-ASP units (14 of 167, 8.4%) and in ASP units (3 out of 41, 7.3%).

Making accurate diagnoses is one of the main goals of the ASP. Overall, the results of the analysis of the antimicrobial therapy in ASP and non-ASP units reinforce the recommendations in pursuing the collection of culture samples before the start of therapy. In addition, we found that non-ASP units make greater use of empirical therapy at the same severity of the patient’s clinical state and more often resorted to the use of antibiotics when not necessary, i.e., the patient’s clinical state was not suggestive of infection. Indeed, we found a rate of about 50% of unnecessary antimicrobial prescription in non-ASP units.

Similar data come from a study conducted in the United States in 2010–2011 that found at least 30% of antibiotics prescribed in doctors’ offices, emergency departments and hospital clinics were unnecessary [19].

In the present study, another factor independently associated with appropriateness in antimicrobial prescription was the presence of bloodstream infections or bacterial endocarditis. These data may be associated with the infectious disease management of bacterial endocarditis and to the structured bacteremia program conducted by infectious disease specialists in ASP and non-ASP units of both hospitals. The infectious disease consultations that were provided to all patients with bloodstream infections have most probably increased the appropriateness of antibiotic prescriptions in this setting and improved the adequacy of patients’ management. In fact, some studies showed that advice from infectious disease specialists reduced inappropriate treatment and resulted in better clinical outcomes in the management of sepsis [20,21] and *Staphylococcus aureus* bacteremia [22,23,24].

Finally, a better prescriptive appropriateness in ASP units was registered even in surgical prophylaxis. One of the strategies of ASP was to optimize surgical antibiotic prophylaxis, ensuring that timely and appropriate antibiotics are administered as recommended before surgery and limiting prolonged use of antibiotic prophylaxis once surgery is over [25]. We found how, not following the guidelines, surgical non-ASP units more frequently used broad-spectrum antibiotics as a first choice for surgical prophylaxis. These findings underline the importance of using a persuasive educational approach that, through audits and drafting of locally adapted guidelines, allows an improvement in the prescribers’ knowledge.

This study has several limitations: even using a study setup that minimized bias inherent in subjective parameter estimation (e.g., the use of standardized treatment guidelines), the experts of infectious diseases may have had motivational biases that influenced their clinical judgment. Moreover, baseline data on the antimicrobial appropriates in wards before the beginning of ASP were not available, making it difficult to compare the outcomes before and after the intervention. Finally, the analysis on the relationship between appropriateness and experience of the physicians was not done. The strengths of our study include its multicenter design and the use of structured intervention that can easily be replicated and incorporated into clinical practice to evaluate the appropriateness of antimicrobial prescription.

## 5. Conclusions

The findings of this study reinforce the importance of adopting an educational ASP in order to improve the quality of antimicrobial prescription in clinical practice and possibly to contribute to a reduction in the global phenomenon of antibiotic resistance. Structured and persuasive interventions aimed at improving prescriber knowledge and compliance, and at optimizing the management of complicated infections such as bacteremia, are of fundamental importance for the success of ASP programs. The efforts of future research should focus on identifying the best strategies to implement an effective stewardship program in hospital settings in order to reduce the misuse of antibiotics.

## Figures and Tables

**Table 1:** Characteristics and global adequacy of antimicrobial prescription between ASP and non-ASP units.

Variables	ASP	Non-ASP	*p* Value
*Characteristics of Units*			
Units N	15	38	−
Beds available N	184	426	−
Patients admitted/ Available beds n/N (%)	117/184 (63.6)	369/426 (86.6)	−
Medical Units n/N (%)	5/15 (33.3)	14/38 (36.8)	0.81
Surgical Units n/N (%)	6/15 (40)	19/38 (50)	0.51
Intensive Care Units n/N (%)	4/15 (26.7)	5/38 (13.2)	0.24
*Characteristics of antibiotic prescriptions*			
Antibiotic prophylaxis n/N (%)	14/117 (12)	64/426 (17.3)	0.17
Antimicrobial therapy n/N (%)	27/117 (23.1)	103/426 (27.9)	0.30
*Prescriptive appropriateness*			
Appropriate antimicrobial prescription n/N (%)	27/41 (65.8)	38/167 (22.7)	<0.001
Inappropriate antimicrobial prescriptions n/N (%)	14/41 (34.2)	129/167 (77.2)	
*Reason for inappropriate antimicrobial prescription*			
Antimicrobial unnecessary n/N (%)	3/14 (21.4)	50/129 (38.7)	0.14
Antimicrobial inadequate n/N (%)	3/14 (21.4)	14/129 (10.8)	0.30
Antimicrobial not recommended n/N (%)	4/14 (28.6)	46/129 (35.6)	0.46
Excessive duration n/N (%)	3/14 (21.4)	40/129 (31)	0.36
Inadequate dose n/N (%)	4/14 (28.6)	19/129 (14.7)	0.24
Inadequate way of administration n/N (%)	0/14 (0)	2/129 (1.5)	0.62
2 reasons for inappropriate antimicrobial prescription n/N (%)	3/14 (21.4)	35/129 (27.1)	0.53
≥3 reasons for inappropriate antimicrobial prescription, n/N (%)	0/14 (0)	3/129 (2.3)	0.55

**Table 2:** Factors associated with appropriate or inappropriate prescription.

Variables	Appropriate Prescription	Inappropriate Prescription	*p* Value
N° of Antimicrobial prescriptions	65	143	−
Antibiotic prophylaxis, N (%)	16 (24.6)	62 (43.4)	0.01
Antibiotic therapy, N (%)	49 (75.4)	81 (56.6)	0.01
Prescriptions in non-ASP units, N (%)	38 (58.5)	129 (90.2)	<0.01
Prescriptions in ASP units N (%)	27 (41.5)	14 (9.8)	<0.01
Characteristics of Units			
Medical units, N (%)	33 (50.8)	41 (28.7)	<0.01
Surgical units, N (%)	22 (33.8)	94 (65.7)	<0.01
Intensive care units, N (%)	10 (15.4)	8 (5.6)	0.02
Appropriateness of antibiotic therapy prescription			
Empiric therapy, n/N (%)	25/49 (51.0)	72/81 (88.9)	<0.01
Prescriptions in non-ASP units, n/N (%)	30/49 (61.2)	73/81 (90.1)	<0.01
Prescriptions in ASP units, n/N (%)	19/49 (38.8)	8/81 (9.9)	<0.01
Acquisition of infection			
Community-acquired infection, n/N (%)	30/49 (61.2)	44/81 (54.3)	0.44
Healthcare-associated infection, n/N (%)	5/49 (10.2)	5/81 (6.2)	0.40
Nosocomial infection, n/N (%)	11/49 (22.4)	20/81 (28.9)	0.04
Unknown, n/N (%)	3/49 (6.2)	12/81 (14.8)	0.13
Severity of infection			
Sepsis, n/N (%)	6/49 (12.2)	5/81 (6.2)	0.23
Septic shock, n/N (%)	1/49 (2)	1/81 (1.2)	0.72
Source of infection			
Unknown source, n/N (%)	1/49 (2)	9/81 (11.1)	0.06
Pneumonia, n/N (%)	9/49 (18.4)	20/81 (24.7)	0.40
Endocarditis/Cardiovascular, n/N (%)	5/49 (10.2)	0/81 (0)	<0.01
Abdominal, n/N (%)	1/49 (2)	15/81 (18.5)	<0.01
Genito-urinary, n/N (%)	6/49 (12.2)	13/81 (16)	0.55
Osteoarticular, n/N (%)	3/49 (6.1)	1/81 (1.2)	0.12
Skin and soft tissues, n/N (%)	5/49 (10.2)	4/81 (4.9)	0.25
Central Nervous System, n/N (%)	1/49 (2)	1/81 (1.2)	0.72
Bloodstream, n/N (%)	9/49 (18.4)	2/81 (2.5)	<0.01
Other, n/N (%)	7/49 (14.3)	7/81 (8.6)	0.31
Missing data, n/N (%)	4/49 (36.4)	7/81 (63.6)	0.92
Collection of microbiology specimen, n/N (%)	37/49 (75.5)	36/81 (44.4)	<0.01

**Table 3:** Logistic regression analysis for independent factors related to prescriptive appropriateness.

Factor	OR	95% CI		*p* Value
Lower Limit			Upper Limit	
Medical units (ref.) Surgical units Intensive care units	0.50 1.70	0.12 0.37	2.04 7.79	0.33 0.49
ASP (ref.) Non-ASP	0.21	0.05	0.85	0.03
Community-acquired infection (ref.) Healthcare-associated infection Nosocomial infection	0.68 0.42	0.11 0.12	4.13 1.53	0.674 0.19
N^o^ sepsis (ref.) Sepsis Septic shock	1.89 0.12	0.28 0.00	12.88 4.34	0.52 0.24
Other sources of infection (ref.) Bloodstream or Cardiovascular infections/ Abdominal	7.16 0.39	1.19 0.04	43.05 3.73	0.03 0.41
Collection of microbiology specimen (ref) No collection of microbiology specimen	0.42	0.12	1.49	0.18

**Table 4:** Adequacy of antimicrobial therapy between ASP and non-ASP units.

Variables	ASP, N (%)	Non-ASP, N (%)	*p* Value
N of antimicrobial therapy prescriptions	27	103	−
*Antibiotic therapy*			
Empiric therapy, N (%)	10 (37)	87 (84.5)	<0.01
Target therapy, N (%)	17 (63)	16 (15.5)	<0.01
*Acquisition of infection*			
Community-acquired infection, N (%)	16 (59.2)	58 (56.3)	0.78
Healthcare-associated infection, N (%)	4 (14.8)	6 (5.8)	0.11
Nosocomial infection, N (%)	4 (14.8)	27 (26.2)	0.21
Unknown, N (%)	3 (11.1)	12 (13.1)	0.94
*Severity of infection*			
Sepsis, N (%)	2 (7.4)	9 (8.7)	0.82
Septic shock, N (%)	2 (7.4)	0 (0)	<0.01
*Source of infection*			
Unknown source, N (%)	0 (0)	10 (9.7)	0.09
Pneumonia, N (%)	6 (22.2)	23 (22.3)	0.99
Endocarditis/Cardiovascular	4 (14.8)	1 (1)	<0.01
Abdominal, N (%)	2 (7.4)	14 (13.6)	0.38
Genito-urinary, N (%)	5 (18.5)	14 (13.6)	0.51
Osteoarticular, N (%)	2 (7.4)	2 (1.9)	0.14
Skin and soft tissues, N (%)	3 (11.1)	6 (5.8)	0.33
Central Nervous System, N (%)	0 (0)	2 (1.9)	0.46
Bloodstream, N (%)	3 (11.1)	8 (7.8)	0.57
Other, N (%)	1 (3.7)	13 (12.6)	0.18
Missing data, N (%)	1 (3.7)	10 (9.7)	0.31
Collection of microbiology specimen, N (%)	23 (85.2)	50 (48.5)	<0.01
*Prescriptive appropriateness*			
Appropriate antimicrobial therapy prescription, N (%) Inappropriate antimicrobial therapy prescription, N (%)	21 (77.7) 6 (22.3)	34 (33) 69 (67)	<0.01
*Reason for inappropriate antimicrobial therapy prescription*			
Antimicrobial unnecessary, n/N (%)	0/6 (0)	31/69 (44.9)	0.03
Antimicrobial inadequate, n/N (%)	1/6 (16.6)	8/69 (11.6)	0.71
Antimicrobial not recommended, n/N (%)	4/6 (66.6)	21/69 (30.4)	0.07
Excessive duration, n/N (%)	0/6 (0)	15/69 (21.7)	0.20
Inadequate dose, n/N (%)	2/6 (33.3)	14/69 (20.3)	0.45
Inadequate way of administration, n/N (%)	0/6 (0)	1/69 (1.4)	0.77
2 reasons for inappropriate antimicrobial therapy prescription, n/N (%)	1/6 (16.6)	17/69 (24.6)	0.66
≥3 reasons for inappropriate antimicrobial therapy prescription, n/N (%)	0/6 (0)	2/69 (2.9)	0.67

**Table 5:** Adequacy of antibiotic prophylaxis between ASP and non-ASP units.

Variables	ASP, N (%)	Non-ASP, N (%)	*p* Value
N° of antimicrobial prophylaxis prescriptions	14	64	−
***Units***			
General Surgery, n/N (%)	0/6 (0)	7/19 (36.8)	0.19
Urogenital Surgery, n/N (%)	1/6 (16.6)	3/19 (15.8)	0.70
Emergency Surgery, n/N (%)	0/6 (0)	2/19 (10.5)	0.50
Orthopedics, n/N (%)	1/6 (16.6)	1/19 (5.3)	0.23
Head and neck Surgery, n/N (%)	0/6 (0)	4/19 (21.1)	0.33
Thoracic Surgery, n/N (%)	1/6 (16.6)	0/19 (0)	0.03
Abdominal Surgery, n/N (%)	3/6 (50)	2/19 (10.5)	0.01
*Prescriptive appropriateness*			
Appropriate antibiotic prophylaxis prescription, N (%)	8 (57.2)	12 (18.8)	<0.01
Inappropriate antibiotic prescription, N (%)	6 (42.8)	54 (81.2)	
*Reason for inappropriate antibiotic prophylaxis prescription*			
Antibiotic unnecessary, n/N (%)	2/6 (33.3)	14/54 (26.9)	0.74
Antibiotic inadequate, n/N (%)	2/6 (33.3)	4/54 (7.7)	0.05
Antibiotic not recommended, n/N (%)	0/6 (0)	23/54 (44.2)	0.04
Excessive duration, n/N (%)	3/6 (50)	23/54 (44.2)	0.79
Inadequate dose, n/N (%)	1/6 (16.6)	5/54 (9.6)	0.59
Inadequate way of administration, n/N (%)	0/6 (0)	1/54 (1.9)	0.73
2 reasons for inappropriate antibiotic prophylaxis prescription, n/N (%)	2/6 (33.3)	15/54 (28.8)	0.81
≥3 reasons for inappropriate antibiotic prophylaxis prescription, n/N (%)	0/6 (0)	1/54 (1.9)	0.73

## Data Availability

The datasets used and/or analyzed during the current study are available from the corresponding author.

## Supplementary Materials

The following are available online at https://www.mdpi.com/2079-6382/10/3/314/s1, Table S1: Main classes of antimicrobials used in ASP and non-ASP and way of administration.
