# Supplementary material for: Impact of an Education-Based Antimicrobial Stewardship Program on the Appropriateness of Antibiotic Prescribing: Results of a Multicenter Observational Study
Source: Antibiotics (Basel). 2021 Mar 17;10(3):314. doi: 10.3390/antibiotics10030314 (PMC8002962; doi:10.3390/antibiotics10030314)
Supplement: Supplementary file 1 [file antibiotics-10-00314-s001.pdf]

Supplementary Table S1. Main classes of antimicrobials used in ASP and non-ASP and way of administration

Variables	ASP, N(%)	non-ASP, N(%)	P value
Antibiotics			
Aminopenicillin, N° (%)	9 (21.9)	49 (29.3)	0.1
Piperacillin/tazobactam, N° (%)	4 (9.7)	26 (15.6)	0.16
Carbapenem, N° (%)	3 (7.3)	10 (6)	0.93
First-generation cephalosporins, N° (%)	13 (53.6)	17 (10.2)	0.01
Third-generation cephalosporins, N° (%)	3 (7.3)	35 (20.9)	0.01
Macrolides, N° (%)	4 (9.7)	3 (1.8)	0.04
Lincosamides, N° (%)	2 (4.9)	0 (0)	0.01
Aminoglycoside, N° (%)	0 (0)	9 (5.4)	0.09
Quinolones, N° (%)	1 (2.4)	29 (17.4)	0.006
Glycopeptides, N° (%)	6 (14.6)	7 (4.2)	0.059
Oxazolidinone, N° (%)	2 (4.9)	2 (1.2)	0.22
Cotrimoxazole, N° (%)	3 (7.3)	3 (1.8)	0.14
Metronidazole, N° (%)	2 (4.9)	13 (7.8)	0.32
Colistin, N° (%)	2 (4.9)	0 (0)	0.01
Lipopetide, N° (%)	0 (0)	1 (0.6)	0.57
Tygecicline, N° (%)	0 (0)	1 (0.6)	0.57
Azole, N° (%)	1 (2.4)	5 (3)	0.67
Echinocandin/Amphotericin B, N° (%)	0 (0)	3 (2)	0.33
Antitubercolosis Agents, N° (%)	4 (9.7)	0 (0)	<0.001
Way of administration			
Oral, N° (%)	9 (15.5)	29 (13.5)	0.95
Intravenously, N° (%)	49 (84.5)	186 (86.5)	0.1
